# Stress Models of Depression: A Question of Bad Timing

**DOI:** 10.1523/ENEURO.0045-17.2017

**Published:** 2017-04-20

**Authors:** Sarah Delcourte, Ouria Dkhissi-Benyahya, Howard Cooper, Nasser Haddjeri

**Affiliations:** INSERM, Stem Cell and Brain Research Institute U1208, University of Lyon, Université Claude Bernard Lyon 1, 69500 Bron, France

**Keywords:** 5d-FSSM, animal model, depression, forced swimming, rodent, stress

Current antidepressant pharmacotherapy remains unsatisfactory because of its limited adherence and partial therapeutic efficacy. Accordingly, there is a crucial need of validated animal models that fully reflect the nature of the disease and do not only separate facets of the disorder.

We read with great interest the recent publication of Mul et al. (2016) reporting that, although the 5 d forced swimming stress model (5D-FSSM) of depression effectively increased floating behavior for 4 weeks, other depressive-like indexes, such as the sucrose preference test, were unaltered. Hence, they conclude that the 5D-FSSM model lacks “construct or face validity to model human depression.”

**Figure 1.**
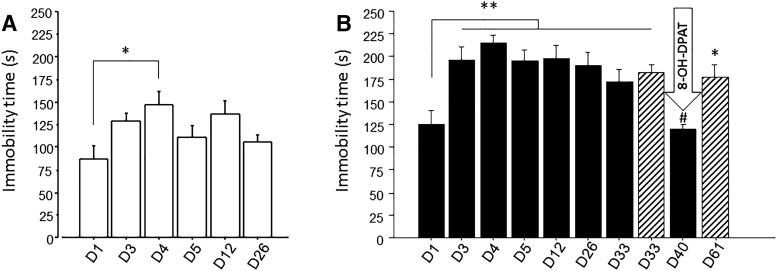
Immobility time in 5d-FSSM. ***A***, ***B***, Six-week-old C57BL/6 mice were forced to swim on 5 successive days (D) for 10 min at ZT1 (***A***; *n* = 6) or ZT 14 (***B***; *n* = 6) following the advice of Sun et al. (2011). Animals were then tested on days 12 and 26. The immobility time was measured over the 10 min of swimming, but only the first 4 min were analyzed. ***A***, Repeated one-way ANOVA revealed an effect of time: *p < 0.01 vs day 1; *post hoc* Tukey–Kramer test showed a difference between D1 and D4. ***B***, On day 40, mice (*n* = 6) received injections of 8-OHDPAT (1 mg/kg, i.p.), a prototypical 5-HT1A receptor agonist, 30 min before the session. Another group (striped bar, *n* = 6) did not receive 8-OHDPAT injection or undergo swim stress on day 40 but was forced to swim again on day 61. Repeated one-way ANOVA showed a main effect of time. **p < 0001, *p < 0.05 vs day 1, #*p* < 0.05 vs D61. Immobility time was significantly shorter on day 1 than on days 3–33. The injection of 8–OHDPAT significantly decreased the immobility time. Results are expressed as the mean values ± SEM.

As these data clearly contrast with those from previous published studies (Sun et al., 2011; Serchov et al., 2015), an obvious question is why? We propose that the study of Mul et al., (2016; as well as certain other studies) fails to take into account a critical factor: the time of day at which the swimming stress tests are conducted. In contrast to the study of Mul et al. (2016), who performed stress tests during the light phase when mice are inactive and normally sleep, we and others (Sun et al., 2011) have found stable depressive-like behaviors when the 5D-FSSM is conducted during the dark phase [zeitgeber time 14 (ZT14)] when nocturnal mice are active, but not at ZT1 during the light (inactive) resting phase (Fig. 1). These data clearly show that the time of stress is crucial for the induction of the depressive-like behavior.

Daily variations in anxiety-like behavior are also observed in rodents. Nakano et al. (2016) showed in C57BL/6 mice a rhythmic expression of anxiety-like behavior with increased anxiety-like behavior in the early part of the dark phase. A recent study (Ben-Hamo et al., 2016) using circadian desynchrony to induce depression like-behaviors in rats further revealed opposite responses of behavioral despair according to whether the forced swim test was performed during the light or the dark phase. Similar chronobiological responses have also been described by Ushijima et al. (2005), who showed that the effects of antidepressants are weak during the light phase but potent during the dark phase. In humans as well, circadian variation of mood is a characteristic feature in depressed patients (Peeters et al., 2006). Hence, appropriate timing is essential to accurately assess anxiety- and depressive-like behaviors in rodents and humans.

The importance of time-of-day (circadian) effects on the etiology of mood disorders and the timing of drug treatments (chronotherapy) as well as of the significant role of clock genes (Landgraf et al., 2014) has been increasingly recognized (Turek, 2016). We thus caution that, depending on the day-night activity pattern of the animal, negative results can be a question of inappropriate timing.
